# An overview of genetic rust resistance: From broad to specific mechanisms

**DOI:** 10.1371/journal.ppat.1006380

**Published:** 2017-07-13

**Authors:** Sambasivam Periyannan, Ricky J. Milne, Melania Figueroa, Evans S. Lagudah, Peter N. Dodds

**Affiliations:** 1 Commonwealth Scientific and Industrial Research Organisation (CSIRO), Agriculture and Food, Canberra, Australian Capital Territory, Australia; 2 Research School of Biology, The Australian National University, Canberra Australian Capital Territory, Australia; 3 Department of Plant Pathology and The Stakman-Borlaug Center for Sustainable Plant Health, University of Minnesota, St. Paul, Minnesota, United States of America; THE SAINSBURY LABORATORY, UNITED KINGDOM

Global agriculture is under threat due to the rapid evolution and spread of pathogenic fungi that cause rust diseases. For instance, the recently evolved races of wheat stem rust (*Puccinia graminis* f. sp. *tritici*) and stripe rust (*P*. *striiformis* f. sp. *tritici*) fungus in parts of Africa, Asia, and Europe are a menace to food security due to their ability to spread rapidly and overcome resistance in common wheat varieties [1]. Similarly, new variants of Asian soybean rust (*Phakopsora pachyrhizi*) detected in Brazil and the United States pose a major constraint to soybean cultivation [2]. Since genetic resistance can provide effective and chemical-free disease control, many efforts are directed towards isolating rust-resistance genes in crop plants and understanding how to best deploy them for durable resistance [3]. In addition, related nonhost species are increasingly being utilised to identify new sources of resistance [4, 5]. Here, we summarise current knowledge of rust resistance, focussing on race-specific, non–race-specific, and nonhost resistance mechanisms.

## Race-specific resistance

**Fig 1:**
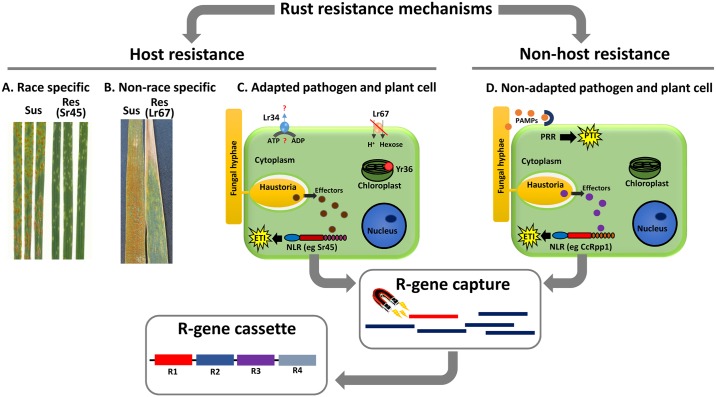
Host and nonhost mechanisms of rust resistance. Race-specific and non–race-specific resistances can be phenotypically quite different (A versus B). **(A)** Strong resistance is conferred by NLR proteins such as stem rust resistance 45 (Sr45) and is associated with a hypersensitive response. **(B)** Non–race-specific resistance may be characterised by partial resistance or slowed fungal growth coupled to leaf-tip necrosis in the presence of genes such as *leaf rust resistance 67* (*Lr67*). **(C)** Adapted pathogens deliver effectors that can subvert pathogen-associated molecular patterns (PAMP)-triggered immunity (PTI), but which may be detected in the plant cell by nucleotide-binding and leucine-rich repeat (NLR) proteins, leading to effector-triggered immunity (ETI). **(D)** PTI can operate in nonhost resistance in response to nonadapted pathogens, and ETI can also occur for pathogens that are more compatible to the host plant. *R*-gene capture methods may be used to detect and identify *NLR* genes, with the goal of incorporation of these *NLR*s into *R*-gene cassettes or stacks to provide durable, long-lasting resistance.

Race-specific resistance genes are effective against some but not all races of a rust pathogen and generally conform to the classical gene-for-gene model, where resistance depends on a specific genetic interaction between host-resistance (*R*) genes and pathogen avirulence (*Avr*) genes. *R* genes in plants predominantly encode nucleotide-binding and leucine-rich repeat (NLR) proteins, which act as immune receptors to recognise pathogen effector proteins delivered into host cells during infection [6]. Much of our understanding of this mode of rust resistance comes from the flax rust pathosystem, in which 19 NLR-encoding *R* genes and 6 corresponding *Avr* gene families have been identified [7, 8]. The flax rust (*Melampsora lini*) *Avr* genes encode small secreted proteins that are expressed in haustoria and recognised inside the host cell (Fig 1C). For at least 2 cases, the recognition event leading to resistance occurs by direct interaction between the flax NLR protein and the *M*. *lini* Avr protein [8]. For the L6 resistance protein, binding to its corresponding effector, AvrL567, favours the active signalling state of the protein in which the nucleotide-binding domain is bound to adenosine triphosphate (ATP) rather than adenosine diphosphate (ADP) [9]. In this state, the N-terminal Toll-interleukin receptor (TIR) signalling domain is thought to be available for oligomerisation events necessary for signalling [10].

Many race-specific rust-resistance genes have been defined genetically in wheat and other crops and are now being cloned in increasing numbers. The recent development of NLR gene capture approaches promises to rapidly expand the repertoire of cloned rust-resistance genes [11]. To date, 9 wheat genes conferring resistance to leaf (*Lr1*, *Lr21*, and *Lr10*), stem (*Sr22*, *Sr33*, *Sr35*, *Sr45*, and *Sr50*), and stripe or yellow (*Yr10*) rust pathogens have been cloned and all encode NLR receptor proteins [3, 11, 12]. The barley *resistance to P*. *graminis 4/5* (*rpg4*/*Rpg5*) stem rust-resistance locus encodes 2 NLRs, which function together as a pair [13], and 1 of these contains an integrated kinase domain, which may act as an effector decoy [14]. Likewise, the wheat *Lr10* locus also includes 2 NLR-encoding genes required for resistance [15]. However, the barley *Rpg1* gene, which confers race-specific resistance to the *Puccinia* stem rust, encodes a protein kinase. Functional studies of Sr33 and Sr50 proteins identified the minimal defense signalling component as the N-terminal coiled-coil domain and showed that dimerisation of this domain is required for signalling [16], similar to observations for the TIR signalling domain in flax NLRs. Knowledge of *Avr* genes outside *M*. *lini* is still limited, but genomics studies are focussing on identifying haustorial or *in planta* expressed secreted effectors and this approach recently identified the first *Avr* gene from coffee leaf rust [17].

## Non–race-specific and multipathogen resistance

As implied, non–race-specific resistance is defined as operating against all races of a pathogen species and is sometimes effective against multiple pathogens [3,12]. Such resistance is generally quantitative, involving a partial resistance phenotype in which the pathogen growth is slowed without an obvious immune response (Fig 1B). In wheat, this resistance is often manifested only at later stages of development and is therefore referred to as adult plant resistance (APR). In contrast to most NLR-encoding *R* genes, some *APR* genes have proved to be highly durable, such as *Sr2*, which has been effective in the field against multiple races of stem rust for almost 100 years [3]. Recent cloning of several wheat *APR* genes has provided some insights into the mechanisms of non–race-specific resistance. For instance, the stripe rust–resistance gene *Yr36* encodes a chloroplast-localised protein with kinase and steroidogenic acute regulatory protein-related transfer (START) lipid-binding domains and is proposed to reduce the detoxification of reactive oxygen species by phosphorylation of a thylakoid-associated ascorbate peroxidase, resulting in enhanced defense responses [18]. The *Lr34* and *Lr67* genes confer APR to several rust and powdery mildew fungi and encode an ATP-binding cassette (ABC) transporter [19] and a hexose transporter [20], respectively. The *Lr67* resistance allele encodes a protein that has lost hexose transport function and could therefore disturb the balance of sugars between the extracellular and intracellular spaces of the leaf. This may reduce the availability of nutrients inside the host cell, hence the effectiveness of this gene against multiple biotrophic fungi. Alternatively, altering apoplastic sugar concentration may induce activation of defense responses [21]. The basis of *Lr34*-mediated resistance and the substrates of this ABC transporter are as yet unknown. In addition to multi-pathogen resistance, both genes also cause a leaf-tip necrosis phenotype associated with accelerated senescence, traits that are also shared with the as-yet uncloned *Lr46* gene. These phenotypic similarities suggest a common mechanism, consistent with the lack of additivity observed when these genes are present in combination. Significantly, genes responsible for race-specific and non–race-specific resistances often do show additivity, supporting their use in concert to achieve stronger protection [3].

Transgenic expression of the wheat *Lr34* or *Lr67* genes in other cereal species, such as durum wheat, barley, rice, and maize, confers resistance to multiple adapted pathogens of these crops, suggesting that the roles of these genes in infection are conserved across a wide taxonomic range [22]. Thus, these genes have the potential to be used as new sources of basal/background resistance in other species, although it remains to be determined whether they can function in eudicots.

## Nonhost resistance

Rust fungi usually have narrow host ranges and poor ability to infect related nonhost species. Nonhost resistance (NHR), exhibited by plant species that do not support full infection by a nonadapted pathogen, offers promise as a source of new genes for crop protection [4]. NHR can result from basic incompatibility when the nonadapted pathogen fails to recognize plant physical and chemical signals necessary for infection. For instance, the wheat stripe rust fungus *P*. *striiformis* f. sp. *tritici* shows a reduced ability to locate stomata in broad bean (*Vicia faba*) [23] and the flax rust fungus *M*. *lini* rarely penetrates rice stomata [24]. In other cases, NHR occurs as a postpenetration event. For instance, *Hemileia vastatrix*, the causal agent of coffee leaf rust, can successfully invade *Arabidopsis thaliana* leaves via stomata but fails to develop haustoria [25], while rice exhibits posthaustorial resistance when inoculated with various cereal rust pathogens (*Puccinia* sp.) [4, 24].

Current models of NHR mechanisms involve a combination of NLR-mediated effector recognition and basal immunity mediated by recognition of pathogen-associated molecular patterns (PAMPs) by cell surface receptors (Fig 1) [26]. Basal immunity would be relatively more important in interactions where the nonhost species is distantly related to the normal host, and NLR immunity more important in interactions involving a more closely related nonhost species. Thus, it is not surprising that many responses associated with NHR overlap those activated during host resistance [4, 27, 28, 29]. For instance, resistance of *Brachypodium distachyon* to different isolates of *P*. *graminis* f. sp. *tritici* differs in strength and timing (pre- or posthaustorial), suggesting race specificity and therefore a role of effector recognition in these phenotypic outcomes [27, 30].

The potential of using NHR as a strategy to identify new rust-resistance traits has been validated with the cloning of *CcRpp1*, an NLR-encoding gene from pigeonpea (*Cajanus cajan*), which confers resistance to the soybean pathogen *P*. *pachyrhizi* [5]. Pigeonpea is closely related to soybean and *CcRpp1* was identified after a screen of accessions displaying phenotypes ranging from partial infection to full immunity. This variation enabled a map-based cloning approach to identify *CcRpp1*, whose expression in soybean conferred resistance to *P*. *pachyrhizi*. Similarly, work towards cloning genetic factors governing NHR against *P*. *striiformis* f. sp. *tritici* is underway, as the resistance locus *Rps6* has now been fine-mapped in barley [31].

## Conclusion

With the rapid emergence and spread of rust pathogens, robust and durable resistant crop cultivars are of immediate necessity to safeguard global agriculture and food production. The propensity of race-specific *R* genes to break down due to changes in pathogen *Avr* genes and the partial resistance conferred by non–race-specific genes means that the most promising deployment strategies involve generating combinations of such genes to minimise the likelihood of pathogen virulence evolution and ensure resistance durability [3]. Such resistance gene pyramids could be developed using conventional breeding approaches using marker-assisted selection based on cloned gene sequences or through the deployment of resistance gene cassettes in which multiple cloned genes may be combined in a single locus (Fig 1). Understanding the potential for additive interactions between resistance genes is important to identify the most effective combinations to pursue, while identifying rust *Avr* genes is also a priority to monitor pathogen evolution and prioritise resistance genes for deployment.
